# Supplementary material for: Copper and Zinc Content in Infant Milk Formulae Available on the Polish Market and Contribution to Dietary Intake
Source: Nutrients. 2021 Jul 25;13(8):2542. doi: 10.3390/nu13082542 (PMC8400833; doi:10.3390/nu13082542)
Supplement: Supplementary file 1 [file nutrients-13-02542-s001.zip › nutrients-1258465-supplementary.pdf]

Table S1. Copper and zinc concentration (mg/100 g powder) in formulae samples

Sample number	Cu mg/100g powder	Producent declaration of Cu mg w 100 g powder	Zn mg/100 g powder	Producent declaration of Zn mg w 100 g powder
		Infant formulae (0-6 months)		
A1	0,36	0,36	4,2	3,9
A2	0,29	0,30*	4,1	3,8*
A3	0,34	0,29*	4,0	3,6*
A4	0,27	0,30*	3,9	3,6*
A5	0,28	0,29	3,9	3,7
A5'	0,29	0,29	4,1	3,7
A6	0,28	0,29*	3,4	3,7*
A6'	0,28	0,29*	4,3	3,7*
A7	0,29	0,31*	5,2	4,7*
A8	0,39	0,38	5,0	5,5
A8'	0,40	0,38	4,8	5,5
A9	0,33	0,35*	4,2	3,9*
A9'	0,34	0,35*	4,2	3,9*
A10	0,30	0,30	4,3	4,2
A11	0,34	0,40	4,0	4,3
A12	0,23	0,30	3,4	3,6
A13	0,33	0,34	5,1	5,1
A14	0,28	0,33	4,4	4,9
A15	0,32	0,33	6,1	5,3
A16	0,35	0,42	3,5	3,7
A16'	0,35	0,42	3,5	3,7
A17	0,36	0,44	4,7	5,3
A18	0,33	0,37	4,2	4,4
A19	0,37	0,42	3,8	3,7
A20	0,35	0,37*	3,9	3,7*
A21	0,37	0,39*	3,7	3,6*
A22	0,35	0,38*	3,8	3,5*
A22'	0,33	0,38*	3,4	3,5*
A23	0,33	0,31*	3,4	3,8*
A24	0,35	0,30*	3,8	3,8*
A25	0,37	0,39*	3,5	3,6*
A26	0,31	0,31	3,3	3,1
A27	0,27	0,30*	3,6	4,4*
A28	0,36	0,40*	3,5	3,1*
A29	0,37	0,42	4,2	4,2
A30	0,43	0,43	5,1	4,3
		Follow-on formulae (7-12 months)		
B1	0,24	0,31	5,1	4,9
B2	0,37	0,27	3,8	3,9
B3	0,26	0,27	3,7	3,7
B4	0,28	0,28	4,3	3,9
B5	0,29	0,29	3,9	3,6
B6	0,31	0,30	4,0	3,5
B6'	0,28	0,30	3,8	3,5
B7	0,31	0,32	5,4	5,0
B8	0,41	0,35	4,7	3,8
B9	0,34	0,36	5,0	4,8
B10	0,25	0,30	4,0	3,6
B11	0,35	0,40	4,3	3,7
B12	0,36	0,33	4,2	4,9
B13	0,24	0,33	5,7	5,2

B14	0,36	0,39	3,8	4,0
B15	0,34	0,37	5,0	5,2
B16	0,34	0,40	3,6	3,7
B17	0,36	0,40	4,6	3,7
B18	0,27	0,27	4,1	4,5
B19	0,31	0,37*	4,2	4,4*
B20	0,40	0,38*	3,7	3,5*
B21	0,37	0,40*	3,9	3,7*
B21'	0,33	0,40*	3,8	3,7*
B22	0,38	0,37*	3,8	3,4*
B23	0,33	0,37	3,5	4,0
B24	0,22	0,27	4,1	3,5
B25	0,26	0,43*	4,8	4,3*
B26	0,36	0,40	4,2	4,0
B27	0,38	0,40	4,6	4,0
B28	0,32	0,32*	3,8	3,5*
B29	0,40	0,38*	3,2	3,5*
Formula for special medical purposes for infants (0-12 months)				
C1	0,27	0,29	3,9	3,8
C2	0,26	0,28	3,2	3,4
C3	0,40	0,30	5,0	4,5
C4	0,40	0,39	6,5	6,0
C4'	0,49	0,39	6,8	6,0
C5	0,29	0,29	4,1	3,7
C5'	0,28	0,29	4,2	3,7
C6	0,29	0,28	3,6	3,5
C7	0,31	0,31	4,3	3,9
C7'	0,33	0,31	4,1	3,9
C8	0,35	0,37	5,2	4,9
C9	0,42	0,38	5,7	5,1
C10	0,45	0,39	6,0	5,2
C11	0,33	0,33	5,4	5,1
C12	0,42	0,45	6,8	6,8
C13	0,32	0,30	5,3	5,0
C14	0,20	0,27	5,0	4,8
C15	0,30	0,95	5,8	5,1
C16	0,28	0,32	3,7	3,6
C17	0,36	0,39	3,9	4,1
C18	0,48	0,41	5,1	5,3
C18'	0,33	0,41	5,2	5,3
C19	0,35	0,38	3,9	3,5
C20	0,36	0,36	5,6	5,3
C21	0,42	0,37	5,3	5,0
C21'	0,47	0,37	5,2	5,0
C22	1,00	0,86	10,1	8,6
C23	0,69	0,77	5,8	6,3
C24	0,36	0,40	4,9	5,0
C25	0,43	0,37	3,7	3,5
C26	0,34	0,38	4,8	4,6
C27	0,34	0,37	5,4	4,7
C28	0,46	0,38	4,1	3,5
C29	0,40	0,41	5,4	3,9
C30	0,37	0,43	6,6	5,7
C31	0,35	0,34	3,7	3,7

' second batch of the same formula

* Calculated based on producent declaration about content element in 100 ml of formula
